# O086: Antibiotic use and vancomycin-resistant enterococcus (VRE) carriage during a large outbreak in a dutch hospital

**DOI:** 10.1186/2047-2994-2-S1-O86

**Published:** 2013-06-20

**Authors:** J van Balveren, LB van der Velden, HW Fleuren, MH Nabuurs-Franssen, A Voss, T Sprong

**Affiliations:** 1 Medical Microbiology and Infectious Diseases, Nijmegen, The Netherlands; 2 Clinical Pharmacology, Canisius Wilhelmina Hospital Nijmegen, Nijmegen, The Netherlands; 3 Medical Microbiology, University Hospital Nijmegen Medical Center, Nijmegen, The Netherlands; 4 Internal Medicine, Canisius Wilhelmina Hospital Nijmegen, Nijmegen,The Netherlands

## Introduction

In 2012 a large number of Dutch hospitals experienced outbreaks of vancomycin-resistant enterococcus (VRE), including our (560 beds) secondary care hospital.

## Objectives

To examine whether (previous) antibiotic use was associated with VRE-carriage.

## Methods

We studied all patients who were found positive for VRE carriage (rectal swab) by PCR and or culture during admission from an outbreak in a large (560 beds) secondary care hospital in the Netherlands, in the period May to August 2012. From these 120 patients antibiotic use was retrieved from the database of our electronic prescription system.

## Results

From the 120 patients identified as VRE carrier, 112 (93,3%) had used one or more antibiotics in the 3 months before VRE-positivity. 78 (69.6%) patients had used ciprofloxacin and 52 (46.6%) a third-generation cephalosporin (ceftriaxone or cefotaxime). Interestingly, only 1 patient (0.9%) had used vancomycin in the 3 months before positivity. Median duration of admission and time to VRE-positivity were significantly longer in the patients who had received prior antibiotic therapy (14 days) compared to the patients who did not receive antibiotic therapy (4 days).

## Conclusion

VRE-carriage is almost exclusively seen in patients who have previously received antimicrobial therapy. The short time to positivity in the patients who did not receive previous antibiotic therapy may suggest that they acquired VRE in the community or during an admission > 3 months before VRE positivity. In this outbreak, vancomycin use was not related to VRE carriage. A retrospective case control study will be performed on these patients to identify which antibiotics predispose to VRE-carriage.

## Disclosure of interest

None declared.

